# Bacterial alteration of redox stressors impacts environmental stability of influenza A virus

**DOI:** 10.1128/msphere.00125-26

**Published:** 2026-04-20

**Authors:** Matthew R. Williams, Hannah M. Rowe

**Affiliations:** 1 Department of Microbiology, Oregon State University 2694, Corvallis, Oregon, USA; The University of Arizona, Tucson, Arizona, USA

**Keywords:** influenza, *Streptococcus pneumoniae*, catalase, transmission

## Abstract

Influenza A virus (IAV) causes annual morbidity and mortality and remains a constant pandemic threat due to the emergence of novel strains. Therefore, understanding the factors important in host-to-host transmission of IAV is a key control point for protecting individual and public health. Transmission is highly heterogeneous with viral factors and host inflammatory and immune factors being implicated. Also implicated is the upper respiratory microbiome. While typically thought to act indirectly on viral pathogenesis, in an immunomodulatory capacity to enhance or reduce susceptibility to viral infection, recent studies on the pathogenesis of IAV have identified direct interactions between the virus and upper respiratory pathobiont bacteria. We hypothesize that the bacterial cells and their metabolites co-shed into respiratory droplets with IAV particles alter the viability of the IAV particles in the environment, therefore altering the capacity for host-to-host transmission. In this investigation, we utilize a simplified model of fomite transmission in the absence of confounding host factors and demonstrate how oxidative stress from both the environment and the metabolic activity of *Streptococcus pneumoniae* contributes to the killing of IAV, while catalase or the metabolic activity of *Staphylococcus aureus* can protect IAV from environmental or pneumococcally produced reactive oxygen species. These findings support a mechanism for bacterial modulation of viral transmission where bacterial metabolic products present in the respiratory droplet are capable of stabilizing and destabilizing viral particles during environmental transit and therefore modulating viral transmissibility.

**IMPORTANCE:**

Influenza A virus is a major cause of illness and death every year. A key knowledge gap exists in understanding what factors modulate viral transmission. One potential mediator of viral transmission is the bacteria that are found in the human nasopharynx. However, the mechanisms responsible for bacterial modulation of viral transmission are unclear. Here, we utilize a simplified model of environmental survival where we expose viral particles to indoor environmental conditions in the presence of bacterial cells. We demonstrate that hydrogen peroxide produced by *Streptococcus pneumoniae* reduces viral environmental survival, while incubation with catalase or viable *Staphylococcus aureus* cells can protect viral particles from *S. pneumoniae*-mediated viability loss. This supports a model of trans-kingdom bacterial-viral interactions where bacterial metabolites produced in the respiratory droplet are capable of modulating viral environmental survival and therefore transmission.

## OBSERVATION

The key to protecting individual and public health is understanding pathogen transmission. One pathogen of great historical and current public health threat is the influenza A virus (IAV). IAV is shed and acquired in the human upper respiratory tract (1) via airborne particles (2, 3) and fomites (4), which can also be sources of re-aerosolization (5). IAV environmental survival is mediated by temperature and humidity (6, 7) as well as the contents of respiratory droplets (8). Here, we propose a role of an additional component of the contents of respiratory secretions: bacterial products produced by commensals of the human upper respiratory microbial community and co-shed into the respiratory droplet.

Microbial modulation of transmission of IAV has been suggested in IAV household transmission cohorts (9) and in animal models (10–12). The suggested mechanisms are immunomodulation, enhanced symptoms increasing shedding from an infected host, and a more receptive tissue environment in the contact. Recently, direct interactions have been reported between IAV and several upper respiratory pathobiont bacteria (13, 14), suggesting an additional mechanism of modulating viral transmission through bacterial components or metabolites stabilizing or destabilizing the adherent viral particles. This is not unprecedented, as bacterial-viral interactions with enteric viruses have been shown to protect the stability of the virions in the environment (15, 16).

**Fig 1:**
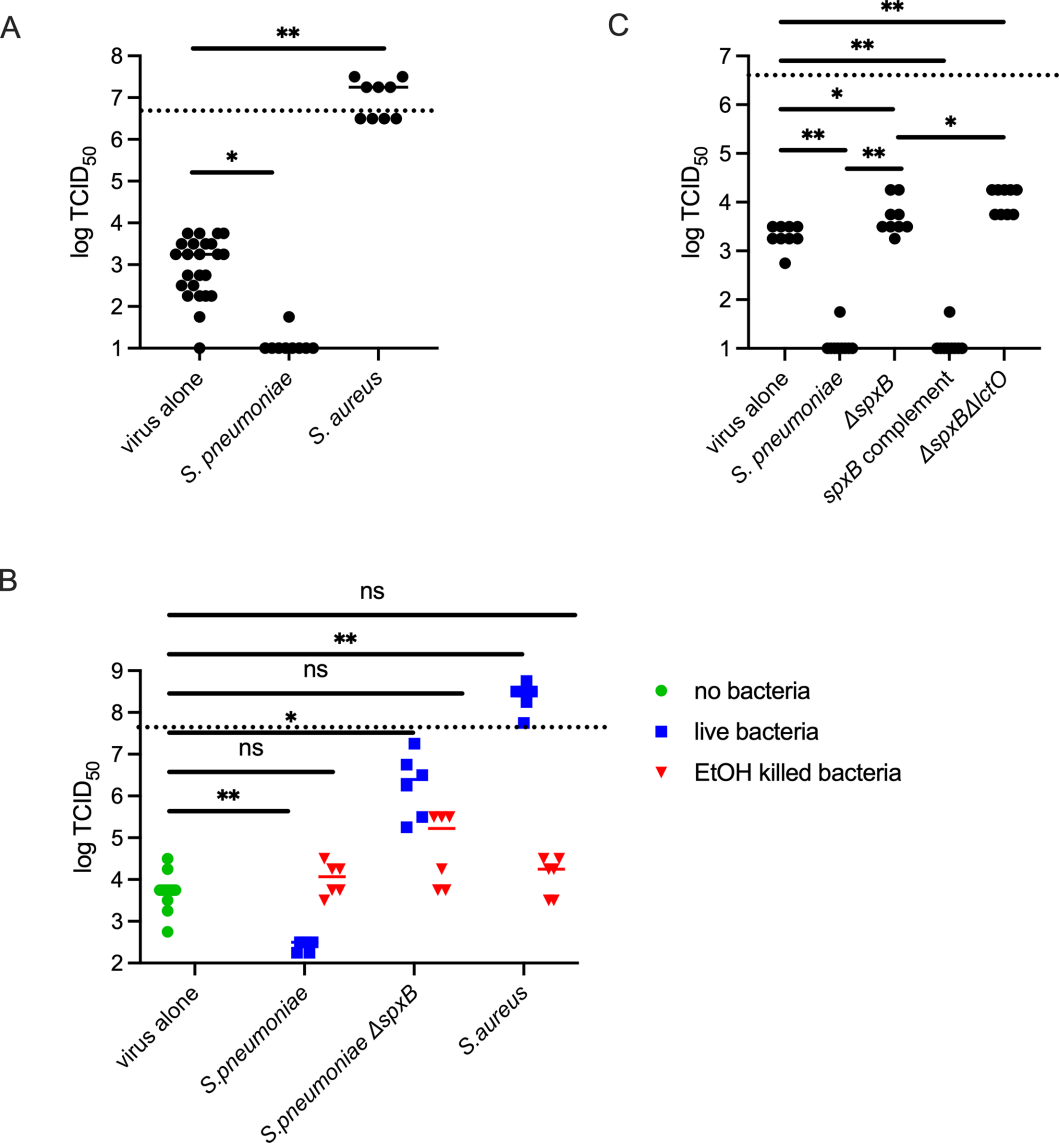
Bacterial H_2_O_2_ production and detoxification mediate viral environmental survival and require live bacteria. (**A**) A total of 5 × 10^6^ TCID_50_ IAV strain PR8 was desiccated at room temperature for 24 h alone or in the presence of 10^7^ CFU of the indicated bacterial strain in PBS. (**B**) A total of 5 × 10^7^ log TCID_50_ IAV strain PR8 was desiccated at room temperature for 24 h alone or in the presence of 10^7^ CFU live or ethanol-killed bacteria of the indicated species. (**C**) A total of 5 × 10^6^ TCID_50_ IAV strain PR8 was desiccated at room temperature for 24 h alone or in the presence of 10^7^ CFU wild-type *S. pneumoniae*, or isogenic mutants of the hydrogen peroxide producing enzymes of *S. pneumoniae*: Δ*spxB*, *spxB-*complement, or Δ*spxB*Δ*lctO* double mutant. Viable virus after desiccation was determined by TCID_50_ on Madin-Darby Canine Kidney cells. Data are plotted with each point representing a single replicate, and the bar is the median. *N* = 6–24 per group. The dashed line indicates the viral titer prior to desiccation. *P < 0.05 and **P < 0.01; ns, not significant by Mann-Whitney.

Previous work demonstrated that *Streptococcus pneumoniae* could protect IAV in a capsule-dependent manner using vacuum and centrifugation for rapid desiccation (11). To model a more biologically relevant desiccation, we mixed viral samples with washed bacterial cultures resuspended in phosphate-buffered saline (PBS) in tissue culture-treated polystyrene well plates at normal indoor conditions for 24 h in the dark, in a total volume of 150 μL. After 24 h, all liquid had evaporated, and the dried material was resuspended in 150 μL of water supplemented with penicillin/streptomycin to kill any surviving bacteria prior to assessing viral viability by 50% tissue culture infectious dose (for full methods, see Supplemental material ). We were surprised to see that *S. pneumoniae*, which improved viral survival in the rapid desiccation protocol (11), in fact reduced survival of IAV (Fig. 1A). Also, *Staphylococcus aureus*, which provided minimal protection of IAV in a model of rapid desiccation (11), was able to significantly increase viral survival compared to virus alone (Fig. 1A), in some cases displaying higher infectivity than the inoculum concurring with prior reports demonstrating that protease (17) and lipase (18) produced by *S. aureus* can enhance IAV infectivity. We suspect that the difference in outcomes between the current desiccation model and the vacuum and centrifugation model (11) is the longer duration of desiccation, gradually over 24 h instead of rapidly over 30 min, allowing more time for viable bacteria to modulate viral survival through metabolic activity, or different timing of metabolite production, changing the balance of protective and damaging products. To determine whether the modulation of viral survival is the result of bacterial metabolic activity or intrinsic to the bacterial cells, we next desiccated the virus in the presence of live or ethanol-fixed bacteria. Prior work demonstrated that ethanol fixation does not alter bacterial-IAV interaction (14). When desiccated in the presence of ethanol-fixed *S. pneumoniae* or *S. aureus*, IAV retained equivalent infectivity to the virus alone (Fig. 1B). This suggests that it is not an intrinsic component, but a product of active metabolism by these bacteria that modulates viral survival.

Previous work demonstrated that Δ*spxB S. pneumoniae* had an advantage in both host-to-host bacterial transmission and in environmental survival of *S. pneumoniae* (19). SpxB, pyruvate oxidase (20), produces hydrogen peroxide (H_2_O_2_) as a metabolic byproduct. As pneumococci do not possess catalase, they cannot detoxify this metabolite and are subject to oxidative damage and loss of viability. Here, we show no loss of IAV viability when desiccated in the presence of Δ*spxB S. pneumoniae* (Fig. 1C), which is reversed in the *spxB*-complemented strain, supporting a role for H_2_O_2_ production by pneumococci in damaging IAV particles and reducing environmental survival. Pneumococcal lactate oxidase, LctO, also produces hydrogen peroxide as a byproduct of metabolism (21). To test the impact of further reducing the ability of the pneumococcus to produce hydrogen peroxide, we next made a strain with both *spxB* and *lctO* deleted that demonstrated significantly higher protection to the IAV particles compared to the *spxB* single deletion (Fig. 1C). In fact, we see enhanced viral survival compared to virus alone when desiccated in the presence of live Δ*spxB* bacteria, suggesting that the metabolic differences induced in this strain (20–23) may be protective to IAV in the environment, supporting a model of pneumococcus producing both stabilizing and destabilizing metabolites. To confirm it is the metabolic differences in the Δ*spxB* mutant and not something intrinsic to the Δ*spxB* mutant cells, as IAV desiccation tolerance is bacterial capsule dependent (11), and Δ*spxB* pneumococci exhibit altered capsule production (21–23), we also tested viral survival when desiccated in the presence of ethanol-fixed Δ*spxB* bacteria and saw no difference between killed wild type and Δ*spxB* (Fig. 1B), suggesting that the lack of metabolic production of H_2_O_2_ is sufficient for the altered viral desiccation tolerance, not the altered capsule levels.

**Fig 2:**
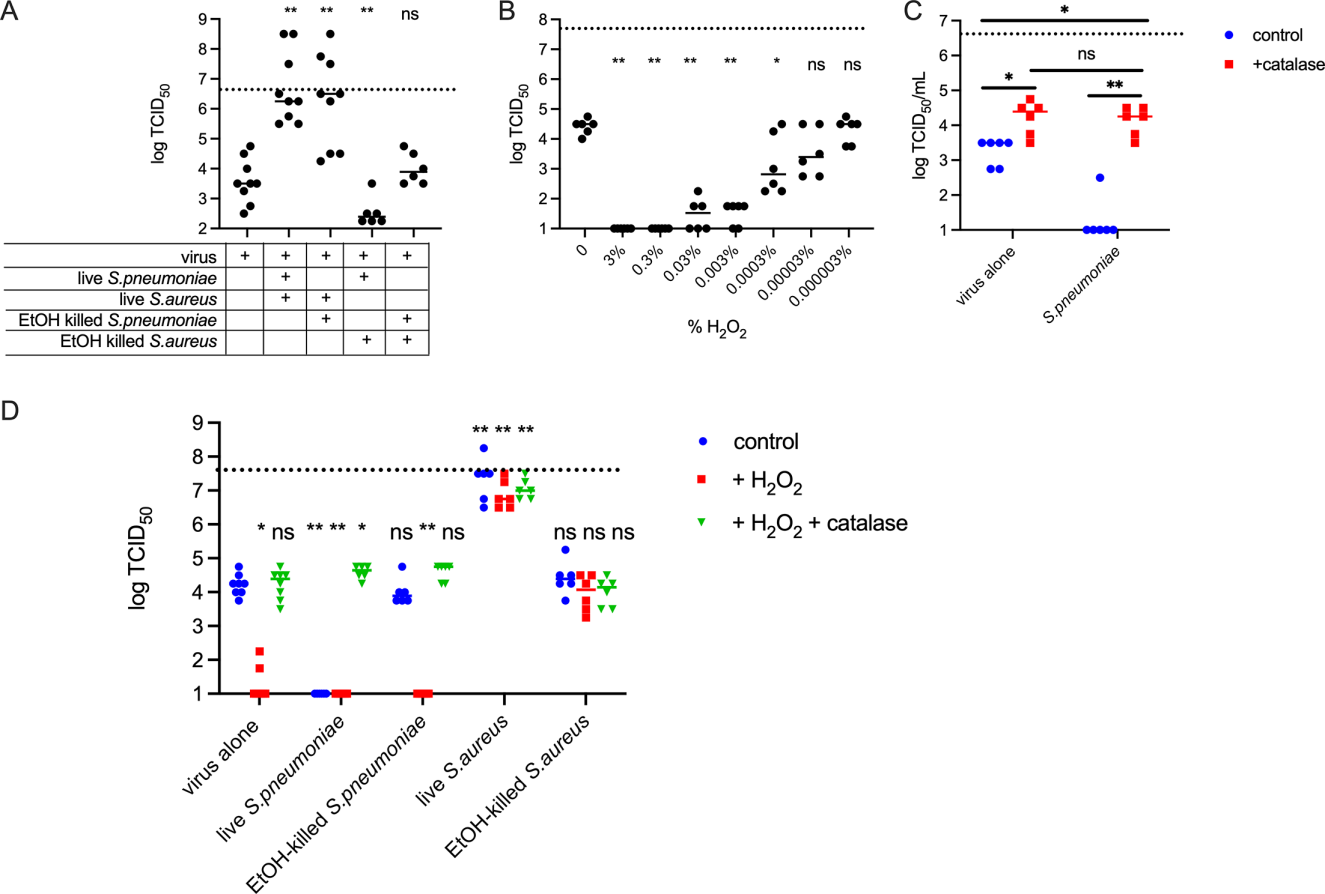
Extracellular catalase activity can protect IAV from pneumococcally generated and environmental ROS. (**A**) A total of 5 × 10^6^ TCID_50_ IAV strain PR8 was desiccated at room temperature for 24 h alone or in the presence of 10^7^ CFU live or dead indicated bacterial strain. (**B**) A total of 5 × 10^7^ TCID_50_ IAV strain PR8 was desiccated at room temperature for 24 h in the presence of the indicated concentration of H_2_O_2_ (**C**) A total of 5 × 10^6^ TCID_50_ IAV strain PR8 was desiccated at room temperature for 24 h alone or in the presence of 10^7^ CFU wild-type *S. pneumoniae*, in PBS (control) or 0.01% catalase. (**D**) A total of 5 × 10^7^ TCID_50_ IAV strain PR8 was desiccated at room temperature for 24 h in the presence of 10^7^ CFU live or dead bacteria supplemented with 0.003% H_2_O_2_ or 0.003% H_2_O_2_ and 0.01% catalase. Viable virus after desiccation was determined by TCID_50_ on Madin-Darby Canine Kidney cells. Data are plotted with each point representing a single replicate, and the bar is the median. *N* = 6–9 per group. The dashed line indicates the viral titer prior to desiccation. *P < 0.05 and **P < 0.01; ns, not significant by Mann-Whitney all compared to virus alone group.

While *S. pneumoniae* does not produce a catalase to detoxify H_2_O_2_, many bacterial species do, including *S. aureus.* We next asked if *S. aureus* could protect IAV from desiccation-mediated viability loss in the presence of wild-type, H_2_O_2_-producing *S. pneumoniae*. When IAV was desiccated in the presence of live wild-type *S. pneumoniae* and live *S. aureus*, IAV particles were protected from viability loss (Fig. 2A), suggesting a role for staphylococcal metabolites in protecting IAV from damaging metabolites produced by *S. pneumoniae*. To determine the role of bacterial viability, we desiccated the virus in the presence of a mix of live and ethanol-fixed *S. aureus* and *S. pneumoniae* cells (Fig. 2A). When live *S. aureus* was present, regardless of if the pneumococci were live or killed, the virus was significantly protected from viability loss compared to virus alone, suggesting that staphylococcal metabolic products can protect IAV. When live pneumococci were present, they required live *S. aureus* to be present for the virus to retain viability, suggesting that a staphylococcal metabolite was necessary for protecting the virus from pneumococcal metabolites, and not merely the presence of staphylococcal cells to protect the virus from pneumococcal metabolites. Similarly, when the virus was desiccated in the presence of killed bacteria from both species, there was no significant difference compared to virus alone, supporting the role of bacterial metabolites in protecting the virus from environmental-mediated viability loss.

To further confirm the role of H_2_O_2_ in viral killing, the virus was desiccated in the presence of increasing concentrations of H_2_O_2_ (Fig. 2B). At any concentration over 0.0003%, viral survival was significantly reduced from the levels seen in the absence of H_2_O_2_. Next, to test the roles of catalase in protecting IAV particles from environmental and bacterially produced reactive oxygen species, we repeated the desiccation protocol in the presence of 0.01% catalase (Fig. 2C). Catalase alone was able to provide significant protection against viability loss. Furthermore, catalase was able to reverse the impact of *S. pneumoniae* and allow the virus to survive in the presence of live pneumococci, supporting our hypothesis that H_2_O_2_ produced by pneumococci is destabilizing IAV. Interestingly, IAV desiccated in the presence of live pneumococci supplemented with catalase demonstrated enhanced survival compared to control virus (Fig. 2C), in alignment with results from other studies demonstrating that pneumococci can enhance viral environmental survival (11, 24), and these findings support a theory that *S. pneumoniae* produces a combination of stabilizing and destabilizing factors that modulate IAV survival.

Next, we tested the combined effect of bacterial viability and exogenous hydrogen peroxide (Fig. 2D). We saw that the addition of peroxide to virus desiccated in the presence of killed pneumococci was sufficient to cause viral killing. Neutralization of peroxide with catalase restored viral survival in the presence of live or killed *S. pneumoniae*, supporting the specificity of hydrogen peroxide killing of IAV. As in Fig. 2C, we again see a suggestion of a factor produced by viable *S. pneumoniae* that promotes viral environmental survival once the masking effect of hydrogen peroxide killing is neutralized by catalase. Peroxide supplementation of samples containing live and dead *S. aureus* did not alter viral survival compared to samples with bacteria and no peroxide or catalase supplementation. For live *S. aureus*, this does fit our hypothesis that catalase production, in addition to other bacterial metabolites, can enhance viral environmental survival, as the bacterial catalase would neutralize the supplemented peroxide, explaining no change in viral viability between the supplemented and unsupplemented groups when live *S. aureus* is present. However, the results from ethanol-killed *S. aureus* do complicate our hypothesis. We would expect that hydrogen peroxide supplementation of killed *S. aureus* would lead to loss of viral survival in the environment. However, we see no change between survival in the presence of killed bacteria alone and supplemented with peroxide. We cannot rule out that residual catalase is present in the killed cells and can neutralize the supplemented peroxide. In Fig. 2A, while the ethanol-killed *S. aureus* are not capable of protection against live *S. pneumoniae*, the survival of the virus is one log higher than we typically saw for virus desiccated with *S. pneumoniae* alone, suggesting a partial protection may have been occurring, and titration of H_2_O_2_ levels may have demonstrated killing of virus in the presence of killed *S. aureus.*

The results shown here demonstrate that metabolically active bacteria can modulate the environmental survival of IAV in both positive and negative ways, with one example being the production and detoxification of hydrogen peroxide during droplet and fomite transmission. This study contrasts with other reports demonstrating a protective effect of *S. pneumoniae* on IAV survival. A recent report demonstrated that in the presence of host mucus, *S. pneumoniae* promotes IAV survival (24). However, catalase is produced constitutively in human respiratory cells (25). Even in the absence of host factors, pneumococci were shown to protect IAV in rapid models of desiccation with vacuum and centrifugation (11) and at early time points in small volumes of saline (26). This suggests that during different transmission modes (quicker drying respiratory droplets instead of longer drying fomites), different mechanisms are likely at play, with pneumococcus being protective in the biophysics of the droplet, preventing salt-supersaturation killing of IAV (27); however, at later time points in larger volumes, slow desiccation allows pneumococcally generated peroxide to accumulate, reducing viral environmental survival. We demonstrate that products produced by viable *S. aureus* are able to enhance IAV environmental survival. Detoxification of H_2_O_2_ is one mechanism but is not sufficient to explain the entirety of enhanced viral survival, which supports a role for other staphylococcal metabolites in promoting viral environmental survival and, therefore, transmission capacity.

## SUPPLEMENTAL MATERIAL

Supplemental Material msphere.00125-26-s0001.docx Full materials and methods.

ASM does not own the copyrights to Supplemental Material that may be linked to, or accessed through, an article. The authors have granted ASM a non-exclusive, world-wide license to publish the Supplemental Material files. Please contact the corresponding author directly for reuse.
