# Supplementary material for: Bacterial alteration of redox stressors impacts environmental stability of influenza A virus
Source: mSphere. 2026 Apr 20;11(5):e00125-26. doi: 10.1128/msphere.00125-26 (PMC13203958; doi:10.1128/msphere.00125-26)
Supplement: Supplemental Material — Full materials and methods. [file msphere.00125-26-s0001.docx]

**Materials and Methods:**

Bacterial cultures:

*S. pneumoniae* strain BHN97 and derivatives were grown from freezer stock on solid media: Tryptic Soy Agar (BD) supplemented with 3% defibrinated sheep blood (Lampire) in a humidified 5% CO_2_ incubator at 37 degrees Celsius. Growth from plates was transferred to THY Broth: Todd Hewitt (BD) + 0.2% yeast extract (VWR), and was grown static in glass tubes in a humidified 5% CO_2_ incubator at 37 degrees Celsius until mid-log, OD_620_ approximately 0.4. BHN97 *spxB* and *spxB-*complemented strain were generated previously(1). BHN97 *spxBlctO* mutant was made for this study (see below.) *S. aureus* strain MW2 (gift from Alex Horswill) was grown from freezer stock in liquid Brain Heart Infusion (BD) (BHI) media overnight at 37 degrees Celsius with aeration. Overnight cultures were diluted 1:200 in fresh BHI and grown at 37 degrees Celsius with aeration until mid-log, OD_620_ approximately 0.4.

Ethanol killed bacteria:

Ethanol killed bacteria were made as previously described(2). Briefly, 10^8^ CFU mid log culture was resuspended in ice cold 70% ethanol and allowed to incubate on ice 5 minutes. Bacteria were pelleted, ethanol was aspirated, and killed bacteria were resuspended in 1 mL PBS. Killing was verified by plating 100μL of killed material on TSA/3% sheep blood and overnight incubation and detection of zero colonies.

Making *spxBlctoO* double deletion:

Allelic exchange with a spectinomycin resistance cassette was performed as previously described(2). About 1kb upstream of *lctO* was amplified from BHN97 genomic DNA using primers (BHN97 LctO up F- GATGGCCCAGTCAACTACC and BHN97 LctO up R spect- TGTATTCACGAACGAAAATCGATAAAATGCCCTCCTTGATTAAGTAAG) and about 1kb downstream of *lctO* was amplified with primers (BHN97 LctO down F spect- GAAAACAATAAACCCTTGCATATGTAAAACAGATTGCCTCCACTGAATG and BHN97 LctO down R- GCGACTGCTTGATTCCAGC) using PrimeStar High Fidelity Polymerase (Takara). PCR products were gel extracted (Qiagen MinElute) and along with the spectinomycin resistance cassette were combined using splicing by overlap extension PCR using PrimeStar High Fidelity Polymerase (Takara). Purified PCR product was transformed into *spxB* deletion strain of BHN97, in THY media supplemented with 0.002% BSA, 0.2% glucose and 0.0002% CaCl_2_ (3) grown to early log (OD_620_ approximately 0.2), diluted 1:100 in supplemented THY, incubated 14 minutes with 2μl/mL each 1mg/mL CSP-1 and CSP-2(4).

Cell culture:

Manin Darby Canine Kidney (MDCK) (ATCC CCL-34) were grown in MEM (Gibco) supplemented with 10% Fetal bovine serum (Cytiva), 1x Glutamax (Gibco), and 1x Sodium Pyruvate (Gibco), in a humidified 5% CO_2_ atmosphere at 37 degrees Celsius.

Viral cultures:

Influenza strain A/Puerto Rico/08/1934 (H1N1) was grown in greater than 90% confluent MDCK cells in Infection Media: MEM (Gibco) supplemented with 1X Gluatmax (Gibco), 0.075% Bovine Serum Albumin Fraction V (Gibco), and 1μg/mL TPCK-Trypsin (Sigma), for 96 hours in a humidified incubator with 5% CO_2_ atmosphere at 37 degrees Celsius. Culture supernatant was clarified by centrifugation at 500 x*g* for 5 minutes and was aliquoted into 0.5mL volumes and stored at -70 degrees Celsius.

Desiccation:

Bacterial strains were grown to mid-log phase and normalized to about 10^8^ CFU/mL in PBS. 50μL A/Puerto Rico/08/1934, 5x10^6^ or 5x10^7^ TCID_50_ (depending on viral stock used) was added to 24 well tissue culture treated plates (Costar). 100 μL bacterial culture, or PBS for virus only controls, was added to each well and swirled to mix. When two bacterial strains were mixed, 50μL of each bacterial strain was used, for a total of 10^7^ CFU and 100 μL volume of bacterial cells. Plates were left open in the dark at room temperature (21-23°C, humidity actively managed) overnight for all liquid to evaporate. Dessicated material was resuspended in 10X suggested working concentration of PenStrep (1000 U/mL Penicillin, 1000 U/mL Streptomycin) solution (Gibco) diluted in sterile de-ionized water and stored at -70 degrees Celsius.

Catalase from Bovine Liver (Sigma) was reconstituted and diluted in PBS so that the final concentration, after addition of bacterial cells and viral culture was 0.01%. 5x10^6^ or 5x10^7^ TCID_50_ (depending on viral stock used) A/Puerto Rico/08/1934 in a volume of 50μL was added to 24 well plates. 10^6^ CFU bacteria in a volume of 50μL, or 50μL PBS for virus only controls, and 50μL of PBS or catalase, was added to each well and swirled to mix, and desiccated and resuspended as above.

30% hydrogen peroxide was diluted in PBS so that the final concentration after addition of viral culture and 10^6^ CFU bacteria, or PBS alone for viral only samples was a total volume of 100μL, and was added to 5x10^6^ or 5x10^7^ TCID_50_ (depending on viral stock used) A/Puerto Rico/08/1934 in a volume of 50μL to wells of a 24 well plate and swirled to mix, and desiccated and resuspended as above.

Determination of viable virus following desiccation:

Rehydrated material was thawed at 4 degrees Celsius. Viral titer was determined by 50 percent tissue culture infectious dose (TCID_50_) assay as described previously(2). Briefly, MDCK cells were seeded at 3x10^4^ cells per well in 96 well plates about 24 hours prior to infection. Serial 10 fold dilutions of rehydrated samples were made in Infection media. Cells were infected in triplicate with each dilution. 72 hours post infection, supernatant from infected cells was mixed 1:1 with 0.5% turkey red blood cells in PBS (Lampire) in V bottom 96 well plates for hemagglutination assay. Plates were incubated at room temperature for 30 minutes. Hemagglutination was read for each well and 50% tissue culture infectious dose was calculated using the method of Reed and Munch(5).

**Statistical analyses**

Statistical analyses were performed using GraphPad Prism Version 10.3.1. Data was tested for normality with the Kolmogorov-Smirnov test and found to have non-normal distribution. Therefore, the Mann-Whitney test was performed for pairwise comparisons.

**References.**

1. Rowe HM, Karlsson E, Echlin H, Chang TC, Wang L, van Opijnen T, Pounds SB, Schultz-Cherry S, Rosch JW. 2019. Bacterial Factors Required for Transmission of Streptococcus pneumoniae in Mammalian Hosts. Cell Host Microbe 25:884-891.e6.

2. Rowe HM, Meliopoulos VA, Iverson A, Bomme P, Schultz-Cherry S, Rosch JW. 2019. Direct interactions with influenza promote bacterial adherence during respiratory infections. Nat Microbiol 4:1328-1336.

3. Bradshaw JL, Rafiqullah IM, Robinson DA, McDaniel LS. 2020. Transformation of nonencapsulated Streptococcus pneumoniae during systemic infection. Sci Rep 10:18932.

4. Pozzi G, Masala L, Iannelli F, Manganelli R, Havarstein LS, Piccoli L, Simon D, Morrison DA. 1996. Competence for genetic transformation in encapsulated strains of Streptococcus pneumoniae: two allelic variants of the peptide pheromone. J Bacteriol 178:6087-90.

5. REED LJ, MUENCH H. 1938. A SIMPLE METHOD OF ESTIMATING FIFTY PER CENT ENDPOINTS12. American Journal of Epidemiology 27:493-497.
